# Bridging the Gap: Pregnancy—And Lactation—Associated Osteoporosis

**DOI:** 10.3390/diagnostics13091615

**Published:** 2023-05-03

**Authors:** Mara Carsote, Maria Roxana Turturea, Ana Valea, Cristian Buescu, Claudiu Nistor, Ionut Florin Turturea

**Affiliations:** 1 Department of Endocrinology, Carol Davila University of Medicine and Pharmacy & C.I. Parhon National Institute of Endocrinology, 011683 Bucharest, Romania; 2 Department of Endocrinology, Dej City Hospital, 405200 Dej, Romania; mariaroxanaturturea@gmail.com; 3 Department of Endocrinology, Iuliu Hatieganu University of Medicine and Pharmacy & Clinical County Hospital, 400347 Cluj-Napoca, Romania; ana74us@yahoo.com; 4 Department of Orthopedics and Traumatology, Cluj Emergency County Hospital, 400347 Cluj-Napoca, Romania; cristibue@yahoo.com (C.B.); ionutturturea@gmail.com (I.F.T.); 5 Department 4—Cardio-Thoracic Pathology, Thoracic Surgery II Discipline, Carol Davila University of Medicine and Pharmacy & Thoracic Surgery Department, Dr. Carol Davila Central Emergency University Military Hospital, 011683 Bucharest, Romania; ncd58@yahoo.com

**Keywords:** fracture, osteoporosis, calcium, pregnancy, lactation, hip, vertebral, teripartide, romosozumab, bisphosphonates

## Abstract

Early diagnosis of pregnancy- and lactation-associated osteoporosis (PLO) is mandatory for a good outcome. Standard care is not a matter of conventional guidelines, rather it requires an individualized strategy while true overall incidence and pathogeny remain open issues. This is a narrative review based on full-length English articles, published between January 2021 and March 2023 and accessed via PubMed (no traumatic fractures or secondary osteoporosis are included). Our case-sample-based analysis included 836 females with PLO (the largest cohort based on published cases so far) through 12 studies and 24 single case reports. Except for one survey, these involved retrospective cohorts of small size (6–10 females/study) to medium size (23–47 women/study), and large cohorts with >50 subjects per study (a maximum of 379). Age of diagnosis: from 24 to 40 years for case reports (most subjects being over 30 and primigravida), while original studies indicated an average age between 31 and 34.18 years. Type of fractures underlined a most frequent vertebral phenotype (a mean of 2 to 5.8 vertebral fractures per patient) versus a most severe non-vertebral phenotype (hip and femoral neck fractures mostly requiring surgery). Potential contributors varied: smoking (1/3–1/2 of subjects), family history of osteoporosis (1/3), heparin and glucocorticoid use in pregnancy, low body mass index (majority of cases), hypovitaminosis D; and (with a low level of statistical significance) anti-psychotic medication, gestational diabetes, lupus, thrombophilia, anemia, in vitro fertilization (1/3 in one study), twin pregnancy, tocolysis with MgSO4, and postpartum thyroiditis. Most remarkably, up to 50% of PLO patients harbor mutations of *LRP5, WNT1*, and *COL1A1/A2* (more damaged form with potential benefits from osteoanabolic drugs); gene testing might become the new norm in PLO. The low index of clinical suspicion should be supported by performing magnetic resonance imaging (gold standard in pregnancy) with DXA (in lactation). Low bone mineral density is expected (Z-score varying from −2.2 SD to −4 SD, unless normal which does not exclude PLO). Bone turnover markers might be useful in individuals with normal DXA, in pregnancy when DXA cannot be performed, and in following the response to anti-osteoporosis drugs. Alternatively, microarchitecture damage might be reflected by DXA-trabecular bone score and high-resolution peripheral quantitative computed tomography. Specific medical interventions are currently focused on teriparatide (TPT) use (3 studies; *n* = 99 females treated with TPT and an additional subgroup of 18 patients from the gene-analysis-based study, thus a total of 117 females) which seems to be the therapy of choice as reflected by these new data: 6–24 months, 20 µg/day, no sequential therapy needed; case selection based on high fracture risk is necessary). The first case using romosozumab was reported in 2022. PAO/LAO remains a challenging condition which is a battle for the wellbeing of two individuals, on one hand, considering maternal-fetal outcomes and taking care of the offspring, but it is a battle for a multidisciplinary team, on the other hand, since a standardized approach is lacking.

## 1. Introduction

Multiple changes of mineral metabolism, also affecting skeletal status, take place during pregnancy and lactation in order to sustain fetal/newborn growth and further milk production [1,2,3].

The physiological cross-talk between hypothalamus–hypophysis–gonadal axes and bone during gestation and breastfeeding might be exceptionally affected in cases diagnosed with pregnancy-associated osteoporosis (PAO) and lactation-associated osteoporosis (LAO), part of the complex panel of premenopausal osteoporosis, displaying dramatic consequences on overall health in females confirmed with fractures (also described as spontaneous, fragility, low-trauma, low-energy, osteoporotic, or stress fractures), thus highlighting the importance of adequate recognition and prompt decision making [4,5].

### 1.1. The Frame of PAO/LAO

In 1955, the first publication to address PAO was produced by Nordin B. and Roper A. [6]. The true incidence remains unknown, probably between 4 and 8 cases per 1,000,000 pregnancies [7]. In general, the timing of PAO/LAO is typically identified within the third trimester, respectively early after delivery (mostly in the first month of lactation), related to the fact that 80% of fetal skeleton-associated calcium content is achieved during the third gestation trimester, while the highest necessity for maternal calcium is registered during breastfeeding period [8].

Two-thirds of females who experience PAO/LAO are in their first pregnancy. Increased parity does not seem to be associated with a higher risk of developing PAO/LAO during the following pregnancies, but prolonged lactation might negatively impact menopausal bone mineral density (BMD), according to some authors [9,10,11]. Moreover, increased parity has been found to be correlated with development of menopausal osteoporosis, especially complicated with lumbar fractures [12,13]. Lactation-related bone loss is a physiological and transient process, although some studies have suggested that BMD recovery in LAO might be delayed and the shift to pre-gestation bone turnover and skeleton status might be disturbed in relation to different contributors [10,14,15,16]. 

PAO- and LAO-related pathogenic events are less well understood. Potential risk factors for this exceptional clinical entity include anomalies of calcium–phosphorus metabolism, including hypovitaminosis D, reduced calcium and vitamin D intake, low peak bone mass, chronic malabsorption, celiac disease, heavy smoking, chronic alcohol use, some genetic anomalies, low body mass index (BMI), reduced physical activity, concurrent secondary causes of bone loss (for instance, anticonvulsant medication, glucocorticoid exposure, and long term use of heparin), and family history of osteoporosis [17,18,19,20,21,22].

No specific link to further development of menopausal osteoporosis in females suffering with PAO/LAO has yet been established; some data have suggested that girls born from mothers with PAO have lower BMD than controls, probably in common with the genetic background of their mothers [23,24]. The MAVIDOS (Maternal Vitamin D Osteoporosis Study) trial showed a higher BMD of offspring by the age of 4 to whom consistent maternal vitamin D (VD) supplementation was provided, VD deficiency being identified in a subgroup of females confirmed with PAO/LAO [25]. In contrast, the ROLO longitudinal cohort showed that BMD of 5-year-old offspring was not correlated with maternal calcium intake and bone turnover markers (BTM) in pregnancy [26].

### 1.2. From Presentation to Current Management

Fracture-associated pain remains the main clinical sign of PAO/LAO. Typically, presentation includes multiple vertebral fractures (VF) and less often hip involvement of different types which may be extremely severe and debilitating. Central BMD is generally more affected at the spine than in the total hip and femoral neck regions, according to postpartum DXA (dual-energy X-ray absorptiometry) evaluation that can be safely performed after delivery. Magnetic resonance imaging (MRI) remains the main imaging tool during gestation, for safety reasons (MRI scan with contrast should be carefully used in selected cases according to general safety criteria). Data on using heel quantitative ultrasound for pregnant females or generally in (menstruating) women of reproductive age are less standardized for assessing the threshold for defining PAO [27,28]. 

BTM might reflect skeletal changes during pregnancy [29]. For example, a higher level of bone-resorption marker (urinary CTX) was found in gestation, in association with lower values of CTX reported under maternal VD supplementation, while CTX in pregnancy is inversely correlated with postpartum BMD [30,31].

Early diagnosis of PAO/LAO is mandatory for a good outcome. Standard care is not a matter of conventional guidelines and protocols, rather of a single case-by-case strategy [32]. Immobilization due to multiple VFs, hip/femoral neck fractures, the use of orthopedic braces, and major surgical procedures (such as vertebroplasty or hip replacement) is part of the specific orthopedic approach. On the other hand, the decision to perform emergency cesarean in PAO is sometimes required based on complex and challenging multidisciplinary considerations. Moreover, LAO requires stopping breastfeeding in order to resume the hypo-estrogenic and hyper-prolactinemic status and milk-associated calcium loss and potentially to reduce the levels of PTHrP (parathyroid hormone-related protein). Calcium and VD supplements represent the accepted consensus regarding medication against PAO/LAO. Specific drugs against osteoporosis such as bisphosphonates (BP), strontium ranelate, denosumab (DEN), teriparatide (TPT), and, since 2022, romosozumab (ROM) are selectively offered to patients after delivery. In general, anti-osteoporotic medication exposure may require 6 months up to a maximum of 5 years (especially BPs) depending on case (if at all) [33,34,35,36]. 

Overall, the maternal-fetal outcomes reflect reduced quality of life due to pain, persistent use of analgesics, disability, immobilization, orthopedic surgery, and the less understood long-term impact of exposing young females to specific anti-osteoporotic drugs. Preterm birth and emergency cesarean affect the newborn in cases when the procedure is selected for a better fracture outcome, impairing maternal care of her offspring, and brining the general implications of early lactation restriction [37].

### 1.3. Aim

This study aims to provide a review of state-of-the-art approaches to PAO/LAO from a multidisciplinary clinical perspective, in the context of the current late COVID-19 pandemic era. 

## 2. Method

This is a narrative review based on published articles considering the following inclusion criteria: publication of data between January 2021 and March 2023; PubMed access; full-length English articles; clinically significant data, either original studies or case reports/series. The respective exclusion criteria were: traumatic causes of fractures in pregnancy and lactation; subjects with well-established pre-conception causes of secondary bone loss/fractures/osteoporosis such as osteogenesis imperfecta, glucocorticoid osteoporosis, or chronic conditions with massive impairment of peak bone mass. 

**Figure 1:**
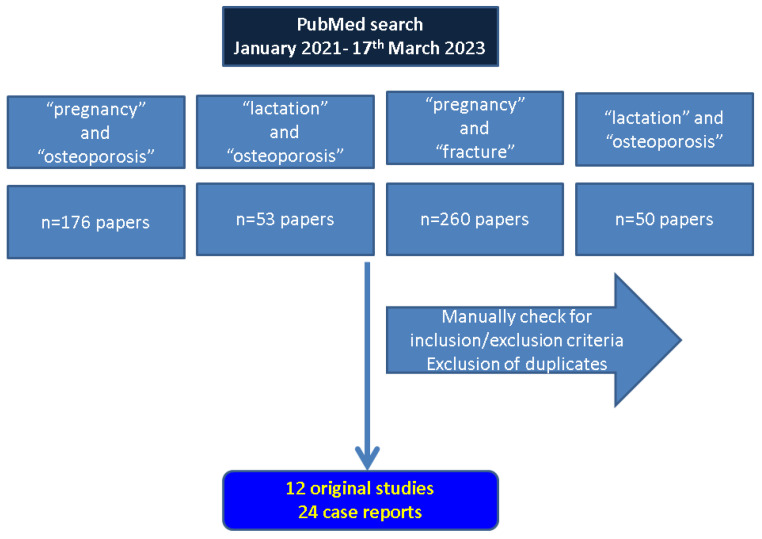
Flowchart according to our methodology (*n* = number of articles).

According to 4 combinations of research key words we identified 176 papers (“pregnancy” and “osteoporosis”), 53 papers (“lactation” and “osteoporosis”), 260 papers (“pregnancy” and “fracture”), and 50 papers (“lactation” and “osteoporosis”). We manually checked each of them, excluded the duplicates and selected 12 studies and 24 case reports (a total of *n* = 836 females with PAO/LAO) according to the inclusion/exclusion criteria. We considered different areas of interest including fracture sites, pregnancy versus lactation onset, original studies versus single case reports, various elements of management from surgery to specific medication against osteoporosis, etc. (Figure 1).

## 3. Results: PAO and LOA

According to our methods, we identified the following 12 original studies (Table 1).

### 3.1. Cohort-Based Analysis 

A nationwide study reported in the Japanese Diagnosis Procedure Combination database included 837,347 females who had 2-year history of obstetric admission (between 2010 and 2014); the rate of fractures within this period of time was 4.5 per 10,000 pregnancies; 6.7% of the cohort had bone imaging or a BTM assay; 7.5% of them had a medication recommendation (considered in this study to be cessation of breastfeeding or administration of specific drugs against osteoporosis). The authors identified potential risk factors for LAO: maternal age older than 40 years, smoking, glucocorticoid exposure, and a high Charlson Comorbidity Index score (of at least 1) [41].

We identified 3 studies on TPT with different perspectives (a total of 99 females treated with TPT in these 3 cohorts). A study including 47 women with PAO/LAO (enrolled between 2006 and 2018) treated with TPT (20 µg/day, 24 months plus VD, followed by stopping any medication against osteoporosis while resuming normal menstruation or taking estrogen–progestin contraceptives) showed an average number of fractures of 4, as confirmed by plane X-ray or MRI. These included incidental fractures under therapy (4/47) that were confirmed within first 3-5 months of TPT (2/4), or after the first 6 months of TPT (2/4). DXA showed statistically significant BMD increases (*p* < 0.001) of 30.1% (lumbar spine), 11.7% (femoral neck), and 12.2% (hip) with similar values after 1 year since stopping TPT [40].

A multicenter, retrospective, 2-year study in females with PAO/LAO treated with TPT (20 μg/day, *n* = 19, median VFs of 4) versus standard care with VD and calcium (*n* = 8 patients, median VFs of 2.5) showed the following: (1-year results) lumbar areal BMD increase of +20.9% (versus +6.2%, *p* < 0.001), total hip areal BMD +10% (versus +5.8%, *p* = 0.43), and (2-year results for 7 versus 6 individuals who completed the 2-year check-up) +32.9% (versus +12.2%, *p* =0.001) for lumbar spine assessments. Moreover, the TBS (trabecular bone score) increase after the first 12 months was in favor of TPT (+6.7% versus +0.9%, *p* = 0.09). P1NP after one month of TPT correlated with lumbar areal BMD after 12 months (*r* = 0.68, *p* = 0.03). No incidental fracture was registered within the study protocol [45].

The third TPT study included 67 women with PAO/LAO (between 2007 and 2017), of whom 43 were assessed at DXA annually for 3 years; among these, 33 females received TPT (a median of 12 months, mean age of 31) followed by anti-resorptive medication (sequential group; N1 = 13, median of 18 months) or drug discontinuation (N2 = 20). Similar lumbar and hip BMD increases were registered after each of the 3 years regardless of the subgroup (N1 versus N2), thus suggesting there is no need for a sequential approach [46].

A single clinical study (between 2010 and 2019) on 10 females (median age of 30 years) showed that 1/10 of them had a twin pregnancy, 4/10 were smokers, and fracture-associated pain was identified at a median of 4 weeks after birth; 7/10 of the subjects had VFs; the mean lumbar T-score was −2.91 SD. Management included stopping breastfeeding (8/10) with VD and calcium supplementation. Pain control was achieved after a mean of 2.4 months (the longest period was in females who continued lactation up to 4 months). One patient required vertebroplasty; four out of the eight females without breastfeeding used BPs, apparently with similar outcomes as seen in BP-negative patients [42].

In addition to a new case report of PAO, Miles et al. introduced a TriNetX-based study (*n* = 135 patients with LAO); the authors took into consideration patients between 10 and 50 years who suffered an osteoporotic fracture within first year after delivery; 44% of the subjects had lumbosacral fractures. Therapy included 10 types of drugs (*n* = 70 patients with available data) at equal rates (of 7%): DEN, TPT, ZOL, pamidronate, ibandronate, etidronate, alendronate, risendronate, calcitonin, cinacalcet [44].

One retrospective study (between 2007 and 2012) compared subjects with PAO (*n* = 7 primiparous with VFs in 6/7 and TOH in 1/7) during lactation with healthy control females who were breastfeeding; trabecular density as assessed by high-resolution peripheral quantitative computed tomography (CT) was 34% lower than in controls (*p* < 0.01), and the cortical thickness was 22% lower (*p* = 0.01), thus proving that both trabecular and cortical microarchitecture are damaged through PAO [47].

A German multicenter study on 42 individuals with PAO/LAO (between 2013 and 2019) identified genetic variants concerning *LRP5*, *WNT1*, and *COL1A1/A2* genes in 21/42 females. The subgroup harboring genetic anomalies had a higher number of VFs (4.8 versus 3.3, *p* = 0.02), while their Z-score was lower (*p* = 0.002) and trabecular and cortical thickness was statistically significantly reduced according to HRPQCT. Among these females, 43% (*n* = 18) received TPT, with a +3.7.7% bone mass increase after 3 years, thus suggesting that females with PAO carrying genetic variants might be candidates for osteoanabolic drugs [48].

One imaging (MRI-based) retrospective study on females (*n* = 1260) older than 15 years and younger than 40 years analyzed compression VFs and identified 6 females with PAO-/LAO-associated VFs, representing 0.5% of the entire cohort. Similar features are found in VFs regardless of the underlying history of PAO/LAO. In this particular instance, the mean age of the subjects was 31 years (between 25 and 37), the average number of VFs was 5.6 (between 1 and 11), and a DXA correspondent with low BMD was reported in 4 out of the 6 subjects [49].

Overall, the mentioned studies display great variation in patient approaches from the perspectives of clinical and imaging evaluation. The patients identified with osteoporosis in pregnancy were typically in their 30 s and were admitted for local pain and functional impotence. Most reports of imaging scans are related to the use of DXA after delivery. 

### 3.2. Transient Osteoporosis of the Hip (TOH) 

TOH, a self-limited condition of late pregnancy or the early postpartum period (but rare reports of TOH in children and middle-aged men are also found) of mostly unknown cause, represents a major cause of dramatic hip pain during this time. MRI is the diagnostic method of choice during pregnancy and, once confirmed, it becomes a comorbidity that should be taken into consideration when deciding the timing and type of delivery (some consider TOH as an indication of emergency cesarean) [38,50,51,52,53,54]. For example, one case of TOH reported a bilateral femoral neck fracture during week 34 of gestation; emergency caesarean was provided to the 24-year-old patient followed by bilateral closed reduction and internal fixation (dynamic hip screws), followed by hip replacement on the left side due to fixation failure. Of note, postpartum DXA assessment showed a Z-score of −0.7, while BMI at the moment of fracture was 32.8 kg/sqm (a value of BMI that is uncommon in PAO) [55].

The true incidence of TOH remains less understood, most cases being unilateral, while bilateral involvement is less frequently reported [55]. The risk factors panel overlaps with general data on PAO/LAO, yet this is still a matter of debate. For instance, one female was recently identified with displaced femoral neck fracture in week 35 of gestation, with reports noting that she had a history of thrombophilia (factor V Leiden) and anemia [56].

TOH-associated pain brings a rather low index of suspicion, and no standard management is recommended; a conservative approach in late pregnancy should be firstly advised after careful consideration by a multidisciplinary team. Postpartum spontaneous recovery is reported within an average of 6 weeks [38,50,51].

A single-center study involving 34 females (mean age of 34.18 y) with PAO (*n* = 17) and LOA (*n* = 17) and unilateral or bilateral (25%) TOH were confirmed via MRI; their profile showed the following potential contributors to TOH: maternal (family) history of osteoporosis in 29% of cases, smoker rate of 47%, IVF (in vitro fertilization) in 32% of cases. Similar maternal outcomes were registered regardless of the type of delivery (cesarean or vaginal) [38] (Table 2).

While the vertebral phenotype is the most frequent, the most severe presentation involves TOH. In this particular situation, DXA evaluation might not reflect the severity of the condition. 

### 3.3. Stress Sacral Fractures (SSF) 

In addition to VFs, non-VF sites also include pelvic fractures [63]. Moreover, SSTs, alternatively named sacral insufficiency fractures or fatigue fractures, are commonly found in primary menopausal osteoporosis, but exceptions are reported, as seen in young females diagnosed with PAO/LAO [39,69,70].

A study from 2023 (*n* = 29,291 pregnant females with a delivery registered between 2016 and 2021) identified 0.078% of these females (*n* = 23, 74% of them were primigravidae) as suffering of SSF-associated lower back and pelvic pain in pregnancy (with MRI confirmation); 74% of them had postpartum pain persistence; one-third of this PAO cohort had 25-hydroxyvitamin D (25OHD) levels less than 20 ng/mL [39].

In 2021, a new case report of a 39-year-old female introduced SSF as part of the PAO panel. The authors reviewed the literature with regard to SST-PAO/LAO and found that 65% of cases are identified during lactation, no particular risk factors have been confirmed, and lumbar radiculopathy is diagnosed in 17.6% of subjects. As an investigation tool, MRI scan is the primary choice, while DXA might not be particularly helpful since 70% of the females do not present with low BMD. Most cases have a favorable outcome with adequately reduced physical activity [71]. Similarly, another report showed that PAO/LAO-associated VFs might not be associated with postpartum DXA anomalies [72]. SSF in pregnancy was also reported due to excessive weight gain when limited outdoor activities were recommended during the COVID-19 pandemic [73]. However, prior studies have shown that PAO/LAO is exceptionally described in obese patients, which might potentially explain higher postpartum DXA-BMD [74,75].

Of note, SSF belong to a larger panel of conditions and PAO/LAO might have only a small epidemiologic impact. Awareness of this context is therefore important. 

### 3.4. Case Reports of LAO 

The case-sample analysis revealed some interesting scenarios according to publications from within latest 29 months. A 34-year-old female was treated with TPT followed by ZOL (in a recent case published in 2023) with a good outcome registered by central DXA and high-resolution peripheral quantitative CT scans, but reported values remained lower than similar controls at 40 months after delivery. Genetic tests (considering the traditional panel for monogenic osteoporosis) were negative, thus being suggestive of a polygenic involvement in the pathogenic loop of osteoporosis [58].

A 29-year-old subject experienced lower thoracic and lumbar pain with lower limb irradiation that was more intense when walking and decreased when lying down, 6 months after her delivery. MRI scan confirmed lumbar L1-2 kyphosis, scoliosis, and multiple VFs while central DXA revealed a Z-score less than −4 SD; VD levels were adequate as reflected by serum 25OHD of 28 ng/mL. LAO, as indicated by the clinical and imaging assessment, was treated by stopping lactation (via bromocriptine use), oral cholecalciferol (6000 UI/day), calcium, and alendronate (70 mg/week). Check-up at 4 months showed a good outcome [59].

The first case of PAO/LAO treated with ROM was reported in 2022, for a 34-year-old (primigravida) Japanese female experiencing low back pain 4 weeks after birth while she was breastfeeding. Low BMD-DXA (lowest Z-score of −2.2 at the lumbar spine) and multiple VFs at lumbar L1-L4 confirmed LAO. She started TPT (for 4 months, 56.4 μg/week, twice a week) which was stopped because of persistent nausea, but also inefficiency (an incidental VF at T11 was detected); she was therefore offered ROM for 1 year without new fractures (BMD increases of 23.6%, 6.2%, and 11.2% at lumbar spine, femoral neck, and hip) [60].

The subject was a 34-year-old female who experienced back pain unresponsive to usual analgesics, which turned out to be LAO-associated multiple VFs (at thoracic and lumbar level) requiring her to stop lactation and start anti-osteoporosis drugs. Potential contributors, as suggested by Ferreira et al. [61], are systemic lupus and positive anti-phospholipid syndrome [61]. Novel contributors might be part of a polygenic spectrum, as in the case of a 35-year-old woman who experienced multiple VFs two months after her second birth. Her lowest DXA Z-score was of −3.5 SD, and she was identified as carrying a *WNT1* mutation (heterozygote missense - p.Leu370Val). She received TPT for 24 months that increased lumbar BMD by 14.6%, femoral neck BMD by 8.3%, and hip BMD-DXA by 4.9% [62]. Another risk factor for LAO was suggested in the analysis by Lio et al. [64] with respect to long-term tocolysis with MgSO_4_ and associated glucocorticoid exposure (also with a new case report). This procedure (that allows fetal lung development) increases urinary calcium loss and might cause hypocalcemia due to magnesium-induced parathormone (PTH) suppression, thus potentially causing reduced BMD [64]. Han et al. [65] proposed a new pathogenic element for LAO: postpartum thyroiditis associated with thyroid hormone flare-up. A first such case was reported in 2021: a 25-year-old female was diagnosed with multiple VFs after delivering her first child. She stopped lactation, daily calcitriol (0.5 μg) and calcium (1 g) were added to ibandronate 3 mg/3 month during rehabilitation, and thyroid profile progressively improved [65]. Another 33-year-old female was confirmed with LAO (VFs at lumbar L1 and L4 levels) after a pregnancy requiring heparin use; DEN (60 mg every 6 months, subcutaneous) was offered for 18 months with a DXA-BMD increase of +32.2% (lumbar), +13% (femoral neck), +11.5% (total hip); she later had a second pregnancy and her BMD value, while being decreased, never reached the nadir experienced previously [66].

### 3.5. Case Reports Requiring Surgery 

While VFs are more frequent than hip involvement, vertebroplasty is less reported than surgery for hip and femoral neck fractures due to their severity [42]. For instance, a 38-year-old female had an apparently spontaneous femoral neck fracture as part of PAO in week 28 of her gestation; she presented a sub-capital femoral neck fracture that required closed reduction and internal fixation in a surgical procedure that went well. After cesarean (week 38), she was followed for 2 more years after fracture consolidation with a good outcome [76]. 

Another 37-year-old woman suffered a hip fracture (gestation week 38); MRI confirmed this particular type of TOH. After emergency cesarean was performed and positron emission tomography–computed tomography (PET-CT) scanning excluded other causes of fractures, total hip arthroplasty was performed. Of note, 25OHD was low (25 nmol/L, normal levels between 50 and 160 nmol/L), and she further continued with VD supplementation, ZOL, and no breastfeeding [57].

A primigravida in her 30 s was no longer able to walk soon after delivery by cesarean (week 37) due to persistent hip pain (since week 28 of pregnancy). She had a history of schizophrenia and associated drug-induced hyperprolactinemia and was confirmed with gestational diabetes mellitus. X-ray confirmed bilateral sub-capital femoral neck fracture requiring total hip replacement (right) and internal fixation (left) (one-time postpartum surgery) as part of TOH management. Her 25OHD was normal, and her Z-score (according to central DXA) was low, less than −3.7 SD, and thus she was offered BP for 6 months with a good clinical outcome [23]. The earliest LAO onset was reported within the 6^th^ day after birth in a 24-year-old female who was confirmed with displaced sub-capital femoral neck fracture (as a form of TOH) that required hip replacement [68]. Having surgery within the postpartum period, the ability to take care of the newborn might be impaired and the recommendation of continuing breastfeeding is registered in most cases. 

Due to the current level of statistical evidence, the different scenarios we have mentioned according to the case reports might be taken into consideration for personalized decisions until more data become available worldwide. These cases also raise the suggestion of novel potential contributors to bone loss and fractures in young females that have been reported in other forms of osteoporosis, including certain chronic autoimmune or inflammatory conditions or drugs.

## 4. Discussion 

Overall, in addition to 24 new (single) case reports [23,44,51,55,56,57,58,59,60,61,62,63,64,65,66,67,68], we identified 12 original studies (with at least 6 patients with PAO/LAO per study): 1 study was published in 2023 (within the first 3 months of the year), 5 were published in 2022, and 6 in 2021. Except for one survey [43], these were retrospective cohorts (one being a nationwide study in Japan) [41]. The number of patients specifically diagnosed with PAO/LAO varied from small studies (of 6, 7, and 10 females, respectively), to medium sample-size studies (we considered studies enrolling 23, 27, 33, 34, 42, and 47 women, respectively), and large cohorts with more than 50 subjects per study, which included 69, 135, and 379 patients, respectively (a total of 812 individuals with PAO/LAO according to original studies, increasing to 836 when also including the isolated case reports) [38,39,40,41,42,43,44,45,46,47,48,49].

Concerning the types of assessment among the mentioned studies, we found that one study [48] performed a genetic analysis (in addition to two of the case reports [58,62]), another cohort started from a large series of MRI scans [49], and two other studies (and a case report) used high-resolution peripheral quantitative CT [47,48,58]. Two studies analyzed the fracture rate within one year, respectively 2 years since delivery in larger cohorts [41,44]. Remarkably, a maximum of 837,347 women were screened in one of those [41], while another different trial checked for SSFs among 29,291 individuals [39]. 

Specific medical intervention was concentrated around the use of TPT (3 studies; *n* = 99 females treated with TPT and an additional subgroup of 18 patients from the gene-analysis-based study by Butscheidt et al. [48], thus a total of 117 females) which seems to be the therapy of choice as reflected by these new data [38,39,40,41,42,43,44,45,46,47,48,49]. Additionally, Miles et al. [44] showed an equal 7% rate for using 10 types of drugs (10 females per each drug protocol) such as TPT, BPs (ZOL, pamidronate, ibandronate, etidronate, alendronate, and risendronate), DEN, calcitonin, and even cinacalcet (TriNetX-based study) [44].

Regarding the ages of the patients, in case reports these varied from 24 to 40 years (most subjects being within their third decade of life), while original studies indicated an average age between 31 and 34.18 years [38,49]. These data are consistent with prior observations [74].

Of note, we did not restrict our case-sample-based study to a systematic review, since various parameters were differently reported and we opted for a broader perspective. The current work follows a systematic review by Qian et al. [74] from 2021 that included 65 articles (*n* = 338 females with PAO/LAO, but final analysis was performed on 173–191 documented reports depending on end point; the number of cases per article varying from 1 to 107) [74]. All these articles were published between 1990 and 2020. Therefore, we chose to initiate our analysis using reported data from 2021, also taking into consideration the reality of the COVID-19 pandemic which was in its first waves during 2020 (thus necessitating a slightly different methodology). Qian et al. [74] reported the following interesting data: the average age at PAO/LAO diagnosis was 35.7 years (from 19 to 47 years), 91.5% of patients experienced fracture-associated local pain within the last 3-month period of gestation and the first 3-month period of lactation (the earliest from 5 months of pregnancy and the latest to 9 months of lactation) [74].

From our perspective, bridging the gap with respect to PAO/LAO involves a few clusters of elements that are still to some extent open issues under the remarkable umbrella of low statistical evidence due to the rarity of the condition. These elements are discussed below. 

### 4.1. Gap 1: The Time Frame of Defining LAO

As mentioned, typically the diagnoss of PAO/LAO is established by the end of the pregnancy and beginingg of breastfeeding, usually in a 3-month period of each (some consider LAO as being associated with the first year after giving birth) [77,78,79,80]. It is debatable whether a recent history of an obstetric event (within 2 years) might be a part of LAO. Toba et al. [41] identified a fracture rate of 4.5/10,000 pregnancies, which was correlated with contributors including smoking and glucocorticoid exposure within a 24-month interval [41]. We raise the question of whether a recent history of pregnancy and lactation, but not a current status of breastfeeding, might actually underline LAO. Clearly defining lactation status (and even resumption of menses) should be part of LAO diagnosis/differential diagnosis, because a fracture during the bone-recovery phase might not be LAO but may be related to a traumatic event (with less clear data collected during anamnesis) causing a fracture or a secondary form of osteoporosis in young adults. Of note, the majority of maternal calcium loss via milk takes place within the first weeks after delivery. Adjustment of cortical bone in order to allow rapid calcium release from trabecular bone in pregnancy and lactation, despite being physiological, varies greatly from one skeletal site to another and from one person to another [81,82,83].

### 4.2. Gap 2: Re-Shaping the Concept and the Approach of PAO/LOA

The true epidemiology of PAO/LAO remains unknown. Pre-conception prediction of PAO/LAO is impossible [84,85]. A first pregnancy complicated with this type of osteoporosis might not be followed by a second one with the same complications (despite the patient most probably displaying the same list of potential risk factors); on the other hand, experiencing PAO (as a first-time event) during the second pregnancy is described in less than 10–30% of cases [62,66,74].

PAO and LAO are difficult to distinguish from one another; some patients present with fracture-associated pain after gestation, but the actual discomfort started late during pregnancy and remained unexplored. PAO was clearly identified in other females during pregnancy, which persisted according to local examinations or assessments of skeletal anomalies in the postpartum period regardless of VD and calcium replacement (thus PAO might continue as LAO). It is debatable which pathogenic components are more important in inducing fractures in pregnancy or during lactation, whether they are two faces of the same clinical entity with unknown triggers or are actually distinct conditions. Moreover, there is a low index of suspicion regarding TOH as a specific subgroup of PAO/LAO with dramatic severity. Therefore, it is important to obtain adequate diagnosis considering a multitude of differential diagnostics starting from the associated local and general pain.

### 4.3. Gap 3: Vertebral versus Non-Vertebral Phenotype of PAO/LAO

While most cases of PAO/LAO include VFs, non-vertebral involvement includes TOH [38,55,56]. Furthermore, SSFs should be mentioned in this context, as revealed by a large study on SSFs with different causes; a rate of 0.078% is due to this entity, mostly seen in primigravidae [39]. Previous data reported by Qian et al. [74] found that one-third of all fractures were located at thoracic T12 and lumbar L1 and L2 vertebra with an average number of fractures of 4.4 [74]. Our analysis identified an average number of VFs of 4 in 2 studies [40,45] compared with 5.6 in another study [49]. Patients referred for TPT therapy suffered from more VFs than those who were managed with VD and calcium (4 versus 2.5) [42], while the subgroup with genetic variant carriers had higher VFs when compared with the non-genetic subgroup (3.3 versus 4.8) [48]. Rates of incidental fractures after diagnosis of PAO/LAO were established and patients starting anti-osteoporotic drugs were also reported [40,60]. The rate of new VFs under TPT varied from 0% to 7.8% [40,45]. However, this is difficult to attribute to drug inefficiency or to overestimated early effects of those drugs. 

Despite not being a matter of general consensus, malleolar fracture might be part of LAO. For instance, this may apply in the case of a 31-year-old female experiencing such a fracture within the first 3 months since starting breastfeeding; the lesion required open reduction and internal fixation (which was removed one year later while she remained under BPs) [67]. 

Another site yet to be taken into consideration is the tibia [86]. A 25-year-old female was admitted postpartum for bilateral knee pain that turned out to be a stress fracture of the proximal tibia. The event was triggered by restarting physical activity, which should be done carefully [87]. Miles et al. [44] included foot, toe, and ankle fractures in their study, that are debatable in terms of their LAO status; indeed, however, any fracture during lactation should be suspected as being LAO [44]. Finally, we conclude that a general multidisciplinary approach should include assessment of vertebral or non-vertebral phenotype in PAO/LAO, the first being the most frequent, the second one associated with the most dramatic impact in cases with hip and femoral neck fractures; uncertainty remains for all the other sites (if they are actually part of the same condition). 

### 4.4. Gap 4: The Playlist of PAO/LAO Contributors

Obesity is hardly found as risk factor for PAO/LAO due to the fact that increased BMI helps the bone’s load-bearing function and thus bone formation [55,74]. More than 70% of females who experience the condition do not have abnormally high BMI [71]. Low pre-pregnancy BMI might induce a lower BMD after birth [88].

Siva et al. [23] reported the use of anti-psychotic medication, hyperprolactinemia, and gestational diabetes mellitus as contributors to bone loss [23]. Of general note, the panel of gestational diabetes-associated maternal-fetal complications is extremely heterogeneous, and a potential negative skeletal impact might be expected [89,90,91,92].

Ferreira et al. [61] showed that lupus is a potential contributor to PAO/LAO [61]. A retrospective study from 2022 including 30 patients with systemic lupus erythematosus in pregnancy (versus 31 non-pregnant controls suffering the same condition) confirmed postpartum BMD loss compared with controls (*p* = 0.048); the multivariate regression identified that low pre-conception BMI, long-term glucocorticoid exposure, and maternal age at birth were associated with hip BMD loss [88]. Furthermore, thrombophilia (factor V Leiden mutant) and anemia might contribute (single case-report level) to PAO, adding bone issues to a large panel of complications such as increased risk of gestational thrombotic events [56,93].

Toussia-Cohen et al. [38] showed that one-third of females with the condition had family history of osteoporosis, while one-third of them had received an IVF procedure, which seems to be a new player among the contributors, but with an insufficient level of statistical evidence to back up this association [38]. Moreover, half of the women were smokers, smoking being a traditional risk factor for bone loss and increased fracture risk at any age [38,94,95]. Two further studies also confirmed the association with smoker status [41,42]. Aytar et al. [42] reported the condition in twin pregnancy, albeit with an unknown level of statistically relevant correlation [42]. Tocolysis with MgSO4 might aggravate LAO [64], similar to heparin therapy in pregnancy [66]. A first case of postpartum thyroiditis–associated transitory thyrotoxicosis was reported in association with LAO [65], noting the general negative impact of excessive thyroid hormones on bone loss as seen in patients with long-term levothyroxine-suppressive therapy following surgery for differentiated thyroid carcinoma [96]. Hypovitaminosis D was also identified in up to one-third of the LAO population in one study [39,57]. 

All these confirmed previous data (before 2021) showed that the risk of PAO/LOA increases with advanced maternal age (more than half being older than 30 years) and reduced BMI (only exceptional cases are registered in obese patients). One-third of subjects were exposed to anticoagulation medication in pregnancy, one-third of them had positive family history of osteoporosis, while one-fifth were current or past smokers (apparently a lower proportion than in the studies we have identified and mentioned published since 2021) [38,74].

We appreciate genetic testing as the most innovative approach to understanding PAO/LAO pathogenic pathways [58,62]. Particularly, the study by Butscheidt et al. [48] showed that half of these females harbor mutations of *LRP5, WNT1*, and *COL1A1/A2* that are associated with a more damaged bone profile than non-genetic forms, thus making them candidates for osteoanabolic drugs [48]. Probably, testing these genes, especially *LRP5*, could become the new norm for approaching PAO/LAO in the future. 

As previously observed by Qian et al. [74], limited data are provided to determine whether vaginal delivery or cesarean is associatied with higher risk of developing LAO, which remains an open question [74]. 

Furthermore, the increasing prevalence of bariatric surgery in young females of reproductive age might impair bone status in pregnancy and lactation. Generally, a 1–2-year gap between metabolic surgery and pregnancy is recommended [97,98]. We question if bariatric/metabolic surgery should be listed as a potential contributor to PAO/LAO, noting the increasing numbers of young patients benefiting from this procedure (we mention a first published case from 2019) [99], also recognizing that bariatric surgery increases the risk of fracture outside pregnancy, due to malabsorption, VD deficiency, reduced bone formation caused by lower body weight, gut-derivate calciotropic hormones anomalies, etc. [100,101].

A future subject to explore involves pregnancy in transplant recipients, which is reported more frequent nowadays due to a larger younger population that have received this procedure, considering that bone loss might be found in the females of reproductive age (although we have identified no such cases of PAO so far) [102].

In our analysis, we did not include young females with already known causes of fractures such as osteogenesis imperfecta, multiple myeloma, primary hyperparathyroidism, major beta-thalassemia, or glucocorticoid osteoporosis for whom the diagnostic of PAO/LAO is most probably inadequate [103,104,105,106,107]. To the best of our current knowledge, physiological bone loss during late gestation and lactation might decrease the cutoff for fragility fractures in subjects that already have reduced BMD, but PAO/LAO remains distinct from this specific scenario [108]. Of note, Cushing’s syndrome-associated osteoporosis during pregnancy represents an exceptional event that may mimic PAO/LAO [109].

**Figure 2:**
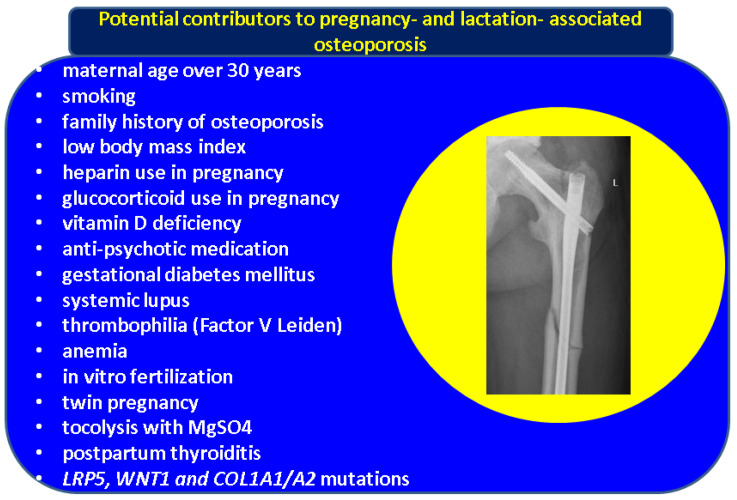
Potential contributors (risk factors) for PAO/LAO (please see the references within the main text; X-ray capture: atraumatic left femoral neck fracture, Garden 4, in a female in her 20 s fixated with 3 femoral neck screws).

Traumatic or iatrogenic (including surgical of different types) injuries in pregnancy should also be differentiated from PAO [110,111,112]. Furthermore, persistent pain at the level of the pelvis after delivery is experienced in one out of six females, and MRI scanning might identify a bone fracture in a very small percent of these in addition to muscle injuries of various kinds [113]. Differential diagnosis of local pain is essential to avoid delaying the fracture identification and decision making to ensure infant care. A survey study involving 69 subjects (members of a social media group of patients who experienced PAO) from 12 countries showed an unexpectedly low rate of 4.4% receiving PAO confirmation within 4 weeks of the local pain starting. TPT was the most used anti-osteoporosis drug, and 6 months after the onset of the fracture-associated pain only 42% of them were able to provide childcare by themselves [43]. Typically, hip (or even lumbar) pain in pregnancy is due to weight gain. In 2021, a novel case of hip avascular necrosis was reported in pregnancy, and the associated pain also might mimic LOA (fewer than 100 cases have been reported so far, according to Mouchantaf et al.) [114]. Another exceptional differential diagnostic concerning back pain involves pregnancy-associated breast cancer metastasis [115]. Postpartum lower abdominal pain might also be caused by chronic symphysitis [116] (Figure 2).

### 4.5. Gap 5: Is There a Place for COVID-19 Pandemic Considerations?

Our research mainly streams the second and third year of the COVID-19 pandemic, whereas medical realities continued to gain attention with the identification ofnew conditions or new approaches to diseases due to the infection itself or social and medical adjustments; overall, all medical and surgical fields have produced dynamic data [117,118,119,120]. Regarding the particular domain of bone changes (including during pregnancy and lactation), reductions in outdoor activities and sun exposure, deficiencies in applying pre-pandemic protocols of serial check-up, chronic stress and anxiety, as well as direct skeletal effects ongoing through coronavirus infection are parameters to be taken into consideration, as seen in the general population [121,122,123]. The infection has not been listed as a potential contributor to PAO/LAO, but two other aspects are reported: one is the decision to perform an X-ray scan in order to confirm PAO during late pregnancy, since MRI scanning was not available due to strict regulations, with a good maternal-fetal outcomes [56]. The second aspect is reflected in the report of a 20-kg weight gain due to self-quarantine in a 40-week pregnant female who experienced an SSF that responded well to a conservative approach [73]. Further data will point out the true lessons we need to learn from this stage of the pandemic era. 

### 4.6. Gap 6: Multidisciplinary Tools to Diagnose PAO/LAO

Clinical suspicion is not enough for a clear diagnosis in most cases of PAO/LAO, but the condition should not be dismissed based on its rarity, because of potential severe consequences that we have mentioned. MRI remains the gold standard for identifying fractures in pregnant females. MRI is allowed during the second and third trimesters, and a case-by-case decision should be made during the first trimester [124,125]. Despite not being a practical alternative, we mention the case reported by Klimko et al. [56] that used X-ray in week 35 (under special circumstances) and no fetal risk was registered [56]. 

A new method (as yet without a current standardized approach) might be represented by the use of blood and/or urinary BTMs. In cases where these are very high, they could be helpful to indicate PAO/LAO in patients with spontaneous fractures, or they should be considered during treatment against osteoporosis [126]. This approach was used amid the COVID-19 pandemic in patients with osteoporosis and associated fractures that were not able to receive medical care in person due to regulations [127]. Lampropoulou-Adamidou et al. [45] pointed out that blood P1NP levels after 4 weeks of starting TPT represent a surrogate marker for predicting the response to the drug by being positively correlated with DXA-BMD after 1 year, as similarly seen in menopausal osteoporosis [45,128,129].

DXA is used only after delivery. We identified two main types of DXA results in LAO: those with normal BMD and low BMD/Z-score. A normal DXA result does not exclude diagnosis when fractures are confirmed and have no alternative cause such as trauma or secondary osteoporosis. However, a normal BMD at periodical check-up is not particularly helpful for patients under specific medication deciding to stop it (and here the use of BTMs might find its place). Some explanations for a normal DXA report might be a high BMI (which is not the norm), an anomoly of microarchitecture rather than BMD, lack of standardization in DXA assessment in the target reference population, local bone loss due to immobilization rather than a general process as found, for instance, in one study where more than two-thirds of subjects with SSFs had normal BMD [71,130,131]. However, a low BMD is generally expected; previous reports showed a mean DXA Z-score of −3.2 SD (lowest value of −7.8 SD) [74]. We found Z-score values varying from −2.2 SD to −4 SD [23,44,51,59,60,62,65].

TBS has been used for exploring bone microarchitecture. No standard TBS interpretation in this population is reported, as opposed to published data on menopausal populations, especially type 2 diabetic females [131]. Moreover, data have shown that grand multiparity is associated with low TBS in the menopause [132]. The use of high-resolution peripheral quantitative CT rather than TBS might support this type of skeleton damage [47,48,58].

Collaterally, we mention that PAO/LAO-induced radiculopathy requires a neurologic evaluation, stressing the importance of having a multidisciplinary team in such cases [71]. 

### 4.7. Gap 7: From “Old” to “New” Therapies for PAO/LAO

Typically, PAO/LAO is self-limited, but its devastating effects such as hip fracture or serial VFs might have a dramatic impact. Once recognized, it is difficult to decide on a drug-free option for patients from the perspective of specific medication against osteoporosis in addition to deciding the type and timing of orthopedic surgery. Due to the rarity of the condition, a team of surgeons and other practitioners should make the decision for hip surgery per primam (or with respect to delivery in PAO) or in cases with an expected poor outcome under medication. 

We observed a dominance of “new” approaches, namely TPT as opposed to prior use of BPs [40,45,46,48,62]. The first use of ROM was reported in a case that was non-responsive to TPT, thus escalating a new alternative line of medication [60]. BPs are used less because they accumulate in bone, but DEN, TPT, and calcitonin display lesser long-term effects so should be considered first [40,45,46,48,62,66]. However, for instance, murine experiments showed that transient use of alendronate during pregnancy and lactation might not be harmful for the newborn [133]. 

Different studies showed a good response and safety profile with regard to TPT in women with premenopausal osteoporosis [134,135]. To our knowledge, three main types of TPT-associated issues remain to be addressed in future: for how long we should treat with TPT; whether sequence therapy (as known from menopausal osteoporosis) is actually necessary; and for how long a consecutive pregnancy (if planned) should be postponed. According to analyzed data, TPT was prescribed from 6 to 24 months (20 µg/day) and a second gestation should be at least 2–3 years after starting TPT [40,45,46,48,62]. Lee et al. [46] revealed that similar results in TPT discontinuation versus a sequential approach were achieved within a 3-year time frame [46]. Hadji et al. [40] also showed that no BPs or DEN are necessary following TPT in order to preserve BMD achieved through TPT exposure, despite the fact that TPT candidates have a more severe osteoporosis profile in terms of multiple VFs [40]. Lampropoulou-Adamidou et al. [46] confirmed that TPT-related BMD gain (mostly lumbar) is higher than with the standard approach using vitamin D and calcium, thus it should be chosen for those patients with a high fracture risk [46]. 

Notably, PAO remains one potential indication for a non-obstetric cause-related cesarean section and a decision should be carefully taken [136].

**Figure 3:**
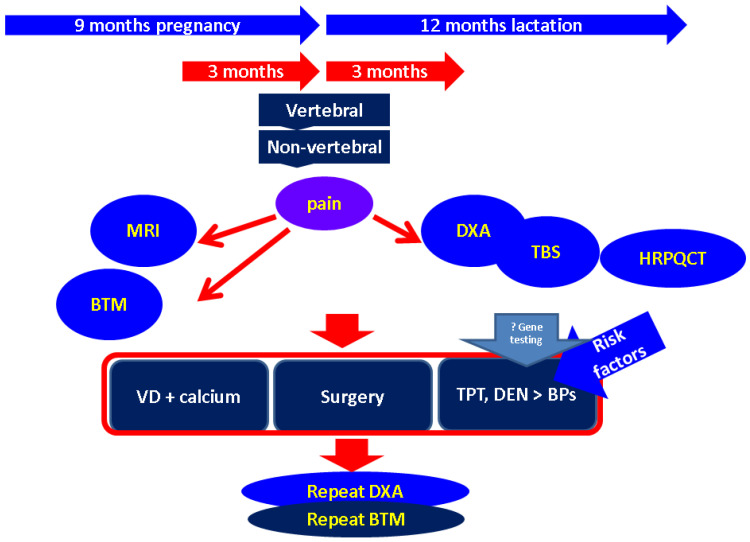
Algorithm for approaching PAO/LAO: starting with local pain due to fractures, the panel of investigation depends on pre-delivery or post-delivery status and is followed by the decision about therapy including standard calcium and vitamin D, surgery (if needed), and medication (in cases with a high fracture risk when considering the potential contributors). Gene testing, particurarly for *LRP5*, *WNT1*, and *COL1A1/A2* genes should be considered in severe cases, probably to help the selection of teriparatide candidates (please see references within main text). Abbreviations: MRI = magnetic resonance imaging; BTM = bone turnover markers; DXA = dual-energy X-ray absorptiometry; TBS = trabecular bone score; HRPQCT = high-resolution peripheral quantitative computed tomography; TPT = teriparatide; DEN = denosumab; BPs = bisphosphonates.

The profile of high fracture risk in PAO/LAO remains an open issue, but we consider that the number of VFs and their clinical impact on the individual are important, in addition to other contributors that we mentioned and which should be taken into account. In the absence of standard risk calculators, a multidisciplinary decision is required in each case. The limits of the current analysis derive from the low level of statistical evidence, the difficulty of approaching such a topic using large randomized trials, variations in the data reported for different cases, the lack of standard protocols, and off-label use of certain anti-osteoporotic drugs (Figure 3).

## 5. Conclusions

PAO/LAO remains a fascinating and challenging condition that requires considering maternal-fetal outcomes and taking care of the offspring, and it is a task for a multidisciplinary team, since a standardized approach is lacking. Personalized medicine represents the basis of this approach. The importance of early diagnosis massively improves the prognosis for mother and her child in both the short and long term. While anti-osteoporotic drugs such as teriparatide or romosozumab might need further studies to be proved helpful and safe, orthopedic intervention from open surgery to conservative management is essential. Longitudinal studies, interventional trials, and larger databases to support genetic diagnosis might represent the next steps in this field. 

## Figures and Tables

**Table 1:** Studies concerning PAO/LAO displayed starting with 2023 (please see references [38,39,40,41,42,43,44,45,46,47,48,49]).

First author Year of Publication Reference Number	Type of Study	Studied Population	Results	Outcome
Toussia-Cohen 2023 [38]	retrospective	*n* = 34 pregnant females with TOH (mean age: 34.18 y)	50% with PAO 50% with LAO	29%: family history of OP 47%: smokers 32%: IVF
Çataltape 2022 [39]	retrospective	*n* = 29,291 pregnant females	0.078% with SSF (*n*′ = 23)	30%: 25OHD < 20 ng/mL
Hadji 2022 [40]	retrospective	*n* = 47 patients with PAO/LAO treated with TPT (20 µg/day, 24 mo)	mean VFs: 4 incidental VF under TPT: 7.8%	After 24 mo (*p* < 0.001): lumbar BMD +30.1% femoral neck BMD + 11.7% hip BMD + 12.2% after 12 mo since TPT (*p* = NS): lumbar BMD + 1.4% femoral neck BMD + 2.6% hip BMD + 4.1%
Toba 2022 [41]	retrospective	*n* = 837,347 females who had 2-year history of obstetric admission	rate of fractures within first 2 y after birth: 4.5/10,000 pregnancies (*n*′ = 379)	7.5%: recommendation for stopping breastfeeding or anti-osteoporotic drugs Other contributors:
				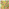 maternal age ≥ 4 y 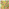 smoking 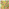 glucocorticoid use 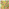 Charlson Comorbidity Index score ≥ 1
Aytar 2022 [42]	retrospective	*n* = 10 females with LAO (median age: 30 y)	LAO median diagnostic: 1 mo 7/10 with VFs	*n* = 1: vertebroplasty Potential contributors:
				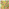 1/10 twin pregnancy 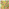 4/10 smoking
Condon 2022 [43]	survey	*n* = 69 females with PAO	4.4%: PAO diagnostic after 1 mo since pain started	42%: were able to provide the child care by themselves after 6 mo
Miles 2021 [44]	TriNetX-based study	*n* = 135 patients with osteporotic fractures within the 1st year postpartum	44%: lumbosacral VFs	Therapy (*n* = 70): each of 7% for DEN, TPT, ZOL, pamidronate, ibandronate, calcitonin, cinacalcet, etidronate, alendronate, risendronate
Lampropoulou-Adamidou [45]	multicenter, retrospective, 2-year study	*n* = 19 patients with PAO/LAO treated with TPT (20 µg/day, 24 mo) *n*′ = 8 patients with VD + calcium	median VFs: 4 versus 2.5	After 12 mo: lumbar aBMD +20.9 versus +6.2% (*p* < 0.001) hip aBMD +10% versus 5.8% (*p* = 0.43) TBS +6.7% versus 0.9% (*p* = 0.09) After 24 mo: N1 = 7 versus N1′ = 6 +32.9% versus +12.2% (*p* = 0.001)
Lee 2021 [46]	retrospective	*n* = 33 patients with PAO/LAO treated with TPT (12 mo) followed by anti-resorptive drugs (N1 = 13, 18 mo) versus none (N2 = 20)	similar age (mean of 31 y) and BMD at baseline (N1 versus N2)	similar lumbar and hip BMD increase after 1, 2, and 3 y (N1 = N2)
Scioscia 2021 [47]	retrospective	*n* = 7 females with PAO	6/7 VFs 1/7 TOH	HRPQCT (versus healthy controls): trabecular density < 34% (*p* < 0.01) cortical thickness < 22% (*p* = 0.01)
Butscheidt 2021 [48]	multicenter	*n* = 42 females with PAO/LAO (genetic analysis)	50% with genetic variants (*LRP5*, *WNT1*, and *COL1A1/A2)*	Females with genetic variants versus non-genetic: higher number of VFs (*p* = 0.02) lower Z-score (*p* = 0.002) HRPQCT: lower trabecular and cortical density
Yıldız AE 2021 [49]	retrospective	*n* = 1260 females with MRI scans	0.5% had PAO/LAO VFs (*n*′ = 6)	mean VFs = 5.6

Abbreviations: aBMD = areal bone mineral density; 25OHD = 25-hydroxyvitamin D = 25OHD; Ca = calcium; DEN = denosumab; IVF = in vitro fertilization; QRPQCT = high-resolution peripheral quantitative computed tomography; LAO = lactation-associated osteoporosis; mo = month; *n* = number of patients; NS = non-significant; OP = osteoporosis; PAO = pregnancy-associated osteoporosis; SSF = stress sacral fractures; TOH = transitory osteoporosis of the hip; TPT = teriparatide; VF = vertebral fractures; VD = vitamin D; ZOL = zoledronate.

**Table 2:** Case reports (one patient per study) concerning PAO/LAO displayed starting with 2023 (please see references [23,44,51,55,56,57,58,59,60,61,62,63,64,65,66,67,68]).

Reference	Case Report	Type of Bone Involvement during Gestation or Lactation Period	Sequence of Specific Anti-OP Therapy/Surgery	Outcome
Bhakta 2022 [51]	40 y	TOH (P1, week 35; hip pain since week 29)	MRI: TOH → conservative approach → elective cesarean (week 37)	femoral neck DXA Z-score= −2.5 SD good clinical outcome at 1 mo (postpartum): no breastfeeding
Klimko 2022 [56]	38 y	TOH (week 35): displaced femoral neck fracture	X-ray (no available MRI amid COVID-19 pandemic): conservative approach → premature rupture of membranes → emergency cesarean → total hip arthroplasty	discharge 5 days after hip surgery potential risk factors:
				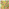 thrombophilia (factor V Leiden) 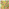 anemia
Varming 2022 [57]	37 y	hip fracture (week 38)	emergency cesarean → hip arthroplasty	→ ZOL
Siva 2022 [23]	34 y	bilateral subcapital femoral neck (P1; postpartum)	total hip replacement (right) + internal fixation (left) (one-time surgery)	→ BP (6 mo)
Treurniet 2023 [58]	34 y	multiple VFs (P1)	TPT → ZOL	postpartum month 40: lower BMD than controls
Jadhav 2022 [59]	29 y	multiple VFs (6 mo after birth)	ALN	postpartum month 4: good clinical status
Kaneuchi 2022 [60]	34 y	multiple VFs (1 mo after birth—G2P2L1)	TPT (4 mo) → ROM (12 mo)	good DXA response under ROM
Ferreira 2022 [61]	34 y	multiple VF	BP	other contributors: SLE, APS
Campopiano 2022 [62]	35 y	multiple VF (2 mo after birth—P2)	TPT (24 mo)	after 24 mo: lumbar BMD +14.6% femoral neck BMD + 8.3% hip BMD + 4.9%
Hourston 2022 [63]	27 y	Two pelvis fractures (P1, 3rd trimester)	elective cesarean at term	
Iio 2022 [64]	36 y	VF (T7 and T12) (G2P1, week 35)	induced labor → vaginal delivery → postpartum DXA, MRI	potential risk factors:
				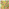 IV MgSO_4_ since week 23 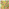 BXM for fetal lung maturation
Miles 2021 [44]	35 y	VF (3rd trimester)	postpartum MRI confirmation	lumbar Z-score = −2.8 SD
Han 2021 [65]	25 y	multiple VFs (1st mo postpartum, G1P1)	IV IBAN 3 mg/3 mo during rehabilitation	femoral neck DXA Z-score = −2.6 SD novel contributor: postpartum thyroiditis
Stumpf 2021 [66]	33 y	VFs—L1 and L4 (3rd mo postpartum; G4P1)	s.c. DEN (60 mg/6 mo, 18 mo)	BMD increase: +32.2% (lumbar) +13% (femoral neck) + 11.5% (total hip)
Wright 2021 [55]	24 y	TOH (bilateral femoral neck fracture)	emergency cesarean → bilateral closed reduction and internal fixation → left hip replacement	good outcome (2-y follow-up)
Bai 2021 [67]	31 y	malleolar fracture (3rd mo postpartum)	open reduction and internal fixation	1 y of BP → removal of internal fixation
Al-Dourobi 2021 [68]	24 y	TOH (6th day after delivery)	hip replacement	good clinical outcome

Abbreviations: ALN = alendronate; APS = anti = phospholipid syndrome; BMD = bone mineral density; BP = bisphosphonates; BXM = betamethasone; DXA = dual-energy X-ray absorptiometry; IV = intravenous; mo = month; OP = osteoporosis; ROM = romosozumab; SLE = systemic lupus erythematous; s.c. = subcutaneous; TPT = teriparatide; TOH = transient osteoporosis of the hip; VF = vertebral fracture; ZOL = zoledronate; y = year.

## Data Availability

Not applicable.

## Abbreviations

BP	bisphosphonates
BMI	body mass index
BTM	bone turnover markers
CT	computed tomography
DXA	dual-energy X-ray absorptiometry
25OHD	25-hydroxyvitamin D
IVF	in vitro fertilization
LAO	lactation-associated osteoporosis
MRI	magnetic resonance imaging
MAVIDOS	Maternal Vitamin D Osteoporosis Study
PAO	pregnancy-associated osteoporosis
PET	positron emission tomography
PTH	parathormone
PTHrP	parathyroid hormone-related protein
ROM	romosozumab
LAO	lactation-associated osteoporosis
SSF	stress sacral fracture
TOH	transient osteoporosis of the hip
TPT	teriparatide
VD	vitamin D
VF	vertebral fractures
ZOL	zoledronic acid
